# Dyskalemia Prior to and After Initiation of a Fixed Dose Combination of Telmisartan and Amlodipine in Adults with Hypertension in Bangladesh

**DOI:** 10.5334/gh.1415

**Published:** 2025-03-19

**Authors:** Junichi Ishigami, Md. Mahmudul Hasan, Aruna Sarker, Sharmin Nahar, Sibly Sadik Shuvo, Mir Ishraquzzaman, Mohammad Abdullah Al Mamun, Md. Kalimuddin, Sheikh Mohammad Mahbubus Sobhan, Di Zhao, Kunihiro Matsushita, Lawrence J. Appel, Sohel Reza Choudhury, Edgar R. Miller, Fazila-Tun-Nesa Malik

**Affiliations:** 1 Department of Epidemiology and Welch Center for Prevention, Epidemiology, and Clinical Research, Johns Hopkins Bloomberg School of Public Health, US; 2 Department of Medicine, Johns Hopkins University School of Medicine, US; 3 Department of Epidemiology & Research, National Heart Foundation Hospital and Research Institute, Dhaka, Bangladesh; 4 Department of Cardiology, National Heart Foundation Hospital and Research Institute, Dhaka, Bangladesh

**Keywords:** Hypertension, Fixed-dose combination, Hypokalemia, Hyperkalemia

## Abstract

**Background:**

The World Health Organization recommends fixed-dose combination (FDC) pills for treating hypertension. Antihypertensive FDC pills often contain a renin-angiotensin inhibitor (RASI) or diuretic. Thus, screening and monitoring for dyskalemia (hypokalemia or hyperkalemia) before and after starting these classes of medications are recommended, a significant barrier for implementation in resource-limited settings. However, the need for blood tests may be overemphasized if the prevalence of dyskalemia in patients with hypertension is uncommon and the incidence of dyskalemia is rare after initiation of FDC.

**Methods:**

We conducted a community-based blood pressure (BP) screening program in Dhaka, Bangladesh, and determined the prevalence of dyskalemia, as defined by K < 3.0 or K > 5.5 mmol/L, in untreated adults with SBP ≥140 mmHg and/or DBP ≥90 mmHg. Among those with a baseline serum K of ≥3.0 or ≤5.0 mmol/L and creatinine clearance ≥30 ml/min, we determined the incidence of dyskalemia 2 months after initiation of a daily FDC of telmisartan 40 mg and amlodipine 5 mg. Secondary outcomes were BP change, medication adherence, and symptoms.

**Results:**

In 2022–2023, we recruited 1,073 adults with SBP ≥140 mmHg and/or DBP ≥90 mmHg. The mean age was 54 years, with 71% men and mean baseline BP 157/94 (SD 12/9.3) mmHg. The prevalence of hypokalemia and hyperkalemia was 1.6% and 0.2%, respectively. FDC was initiated in 1,017 eligible patients, and 864 completed the 2-month follow-up visit. Incident hypokalemia occurred in 1.5% of patients, but there was no case of incident hyperkalemia. The mean change in serum potassium after initiating FDC was –0.05 (0.53) mmol/L. At follow-up, 92% had BP <140/90 mmHg with a mean SBP change of –29.8 mmHg. 1% self-reported mild symptoms (e.g., leg swelling), and there was one death of undetermined cause.

**Conclusions:**

Given low prevalence and incidence of hyperkalemia and evident reduction in BP, our study suggests initiating FDC with telmisartan and amlodipine may be a practical and safe option for newly diagnosed hypertension, especially in resource-constrained settings where blood tests cannot be easily obtained.

## Introduction

Hypertension affects an estimated 1.4 billion people, most of whom reside in low- and middle-income countries (1). Hypertension treatment lowers the risk of cardiovascular disease and mortality by 20–40% (2). However, less than 10% of individuals treated for hypertension are under control in low- and middle-income countries, contributing to substantial morbidity and mortality (1).

In this context, fixed-dose combination (FDC) pills, combining 2 or more classes of antihypertensive medications, have considerable appeal because they improve medication adherence and reduce blood pressure (BP) more effectively than monotherapy (3). The broad use of FDC may also simplify the titration of hypertension treatment and the procurement of antihypertensive medications. Systematic reviews have shown that FDC pills improved adherence by ~30% compared with regimens with separate pills for each drug (4,5).

Most FDC antihypertensive medications include either thiazide/thiazide-like diuretic and a renin-angiotensin system inhibitor (RASI) and sometimes both; it is generally recommended to obtain laboratory testing prior to and after starting FDC to monitor serum potassium levels. However, this requirement for laboratory testing may be an impediment to implementing FDC in low- and middle-income countries where laboratory testing is often unavailable. Further, the need for blood tests after starting FDC may be overemphasized if few patients with hypertension actually develop dyskalemia after the initiation of FDC. For example, a systematic review of randomized trials of RAS inhibitor monotherapy in patients with uncomplicated hypertension showed that the risk of hyperkalemia is low in these patients, and moreover, absolute increases in serum potassium levels are typically small (0.1 mmol/L) (6). However, this systematic review did not include any trials from low- and middle-income countries.

To understand whether laboratory testing is required prior to and after starting FDC in resource-limited settings, we conducted this study to quantify the prevalence and incidence of dyskalemia when FDC medication is initiated in persons with untreated hypertension in Bangladesh. In Bangladesh, where an estimated 21% of adults have hypertension, a major barrier to treating hypertension is the recommendation for periodic laboratory tests (7).

## Methods

### Study design

This study was a single-arm, pre-post design study. We chose this design as it is appropriate for the research question, namely, estimating the incidence and prevalence of dyskalemia with confidence intervals according to the sample size. Also, this design represents a scenario when a clinic starts implementing FDC for a population seeking medical care for newly diagnosed hypertension in Bangladesh. Community-based screening was conducted in the Mirpur area of Dhaka City, Bangladesh (NCT05155436). Mirpur is situated in the northeast part of Dhaka city with a total population of ~1.5 million individuals. Primary objectives of the study were (a) to assess the prevalence of dyskalemia among adults with hypertension, as defined by systolic BP (SBP) ≥140 mmHg or diastolic BP (DBP) ≥90 mmHg, who were candidates for initiating antihypertensive medication; and (b) to assess the incidence of dyskalemia among those who initiated FDC of telmisartan and amlodipine for treating hypertension. We targeted middle-aged and older adults with hypertension, since they represent the largest population segment that would benefit from BP control and, at the same time, be at risk of developing hyperkalemia (8,9). Inclusion criteria were (a) age ≥40 years for men and ≥50 years for women who had menopause for one year (the higher age threshold in women was selected in order to reduce the risk of teratogenicity associated with use of telmisartan during pregnancy); and (b) not currently (in the past month) taking BP medications (Table S1). Exclusion criteria were (a) SBP of ≥180 mmHg or DBP of ≥120 mmHg (e.g., hypertension crisis); (b) history of intolerance or allergy to RASI or calcium channel blocker (CCB); and (c) history of serious medical conditions determined by medical officers (e.g., heart failure, ischemic heart disease, stroke, or chronic obstructive pulmonary disease treated with medications). The protocol was approved by the National Research Ethics Committee of the Bangladesh Medical Research Council (#41419052021) and by the Johns Hopkins School of Medicine Institutional Review Board (#00276132).

### Screening process to identify study population for prevalent dyskalemia

**Figure 1:**
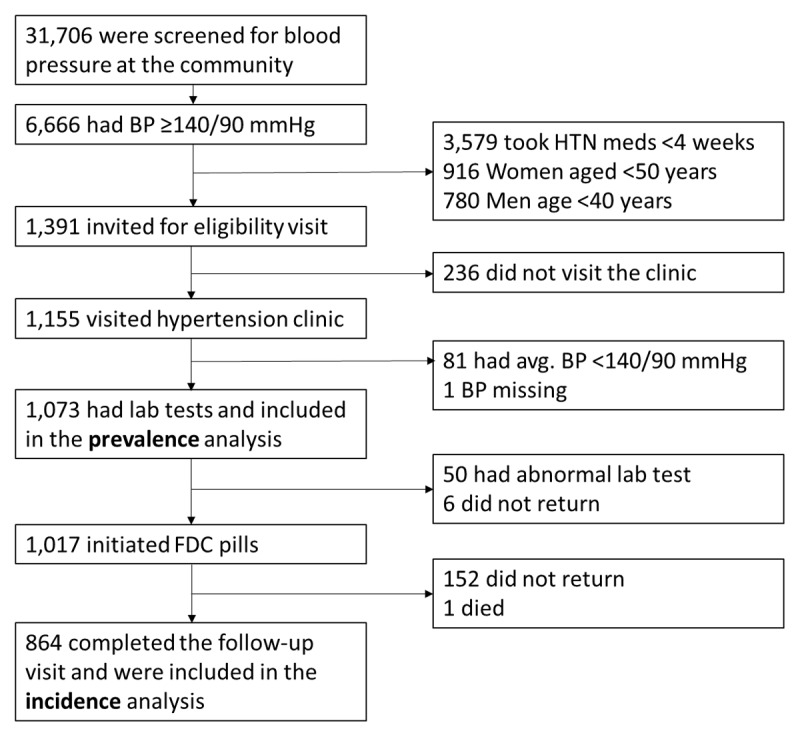
Cohort diagram. Abbreviations: BP, blood pressure; HTN, hypertension; FDC, fixed-dose combination.

After obtaining verbal informed consent, we measured BP for screening, and if participants had an elevated BP (i.e., ≥140/90 mmHg) and met inclusion criteria, they were invited to visit the hypertension clinic in the National Heart Foundation Hospital. At the hypertension clinic, if participants were confirmed to have an elevated BP (i.e., ≥140/90 mmHg) based on an average of the screening and clinic BPs, we collected a blood sample to assess the prevalence of dyskalemia. Between January 2022 and January 2023, we screened 31,706 adults at the community (Figure 1). Of those, 1,391 had BP ≥140/90 mmHg and were not on medication for hypertension; met the other inclusion criteria; and were invited to the hypertension clinic. Of those, 1,155 visited the hypertension clinic, and 1,073 were included in the analysis for the prevalent dyskalemia; each person had visited the hypertension clinic, where a diagnosis of hypertension was confirmed, written informed consent was obtained, and laboratory testing was obtained.

### Study population for incident dyskalemia among those who initiated FDC

Of the 1,073 participants who were included in the analysis for prevalent dyskalemia, we excluded from initiation of FDC those persons with a serum potassium level of >5.0 or <3.0 mmol/L or a creatinine clearance (CrCl) of <30 m/min based on the Cockcroft-Gault equation. We excluded patients with K >5.0 mmol/L from the initiation of FDC since we did not want to count a small change of potassium from 5.1–5.4 to >5.5 as an incident ‘event’. After applying the exclusions, 1,017 participants were provided with FDC pills of telmisartan 40 mg and amlodipine 5 mg and asked to return to the hypertension clinic at 2 ± 1 months for a follow-up visit at which serum potassium was measured (Figure 1). To promote the study participation, our retention strategies included frequent contact attempts by phone or text messaging and offering free transportation to and from the hypertension clinic. After initiating FDC, 152 did not return to the hypertension clinic, and there was one death of undetermined cause. Thus, the analytic sample for the incident dyskalemia consisted of the remaining 864 participants who completed the 2-month follow-up.

### BP measurement procedure

At the screening visit in the community, we first measured BP in a sitting position without resting time. Because the likelihood of hypertension (i.e., BP ≥140/90 mmHg) is low when the first BP is <130/80 mmHg (10), we repeated a BP measurement only when the first BP was SBP ≥130 or DBP ≥80 mmHg. Those who had SBP ≥140 or DBP ≥90 mmHg at the second BP measurement were invited to visit the hypertension clinic. At the hypertension clinic, we first measured BP in a sitting position without resting time, and we repeated a BP measurement only when the first BP was SBP ≥130 or DBP ≥80 mmHg. If the first BP was SBP ≥130 or DBP ≥80 mmHg, the second BP was measured after 5 minutes of rest. A diagnosis of hypertension was confirmed if the average of the two BP measurements at the screening and eligibility visits was SBP ≥140 and/or DBP ≥90 mmHg. For BP measurements at follow-up, BP was measured twice at a single visit, and the average of the two readings was used for the outcome assessment. All BP measurements, both at the screening camp and hypertension clinic, were performed by research staff, who completed BP measurement training for this study and were certified by the principal investigators.

### Intervention

We selected an FDC formulation of telmisartan 40 mg and amlodipine 5 mg (one pill a day) because of its low cost (5.65 cents per tablet as of 2022) and wide availability in Bangladesh. Further, a combination of telmisartan and amlodipine was included in the World Health Organization (WHO) Model List of Essential Medicines (11). Study participants received a three-month supply of FDC pills at the hypertension clinic. The study medication was provided free of charge.

### Outcomes

The primary outcome of interest was prevalent and incident dyskalemia, which was defined by a composite of hypokalemia (K < 3.0 mmol/L) and hyperkalemia (K > 5.5 mmol/L). These thresholds were chosen since potassium levels outside this range often necessitate clinical interventions (e.g., potassium supplementation for hypokalemia (12), RASI discontinuation for hyperkalemia (13)). To make sure that we obtained laboratory data before participants exhausted the three-month supply of FDC pills (i.e., 90 days), we initiated contacts with participants to schedule follow-up visits once 30 days had passed after the initiation of FDC. The primary outcomes of the study were incident hypokalemia and hyperkalemia; secondary outcomes were change in BP, self-reported adherence to the medication, and self-reported symptoms at follow-up. Adherence to the medication was assessed in two questions: ‘How many days did you miss a dose of meds over the past 7 days?’ and ‘When was the last day you took the medication?’ For symptoms at follow-up, we asked whether they had dizziness or lightheadedness or fainting, swelling of ankles, and/or other symptoms.

### Data collection

Data collection was minimized to reduce the burden of study participation and to reflect resource availability in low- and middle-income countries. Age, sex, medical history (heart failure, myocardial infarction, stroke, diabetes, kidney disease, chronic obstructive pulmonary disease, and asthma), smoking status, height, and weight were self-reported. Potassium and creatinine levels were measured in serum samples using the photometry method on a Siemens Dimension EXL with LM fully automated analyzer (Siemens Healthineers, Erlangen, Germany). The level of urinary protein was measured semi-quantitatively using Human’s ‘Combina 3’ urine test strips (HUMAN Diagnostics Worldwide, Wiesbaden, Germany) (reported a none, trace 1+, 2+, or 3+). All sample collection, storage, transport, and laboratory measurements were, performed at a laboratory of the National Heart Foundation in Bangladesh according to standard methods.

### Statistical analysis

Assuming that the observed incidence of dyskalemia would be ~1.5%, we calculated the required sample size such that the upper limit of the 95% confidence interval for the estimate would not exceed 2.5% (prespecified tolerable limit). With a type I error probability of 0.05 (two-sided) and 80% power, the sample goal was 1,290 individuals. However, given that the observed incidence of hyperkalemia (0%) was lower than our assumption, the final sample size of 1,073 was sufficient.

Characteristics of study participants were summarized as mean (SD) for continuous variables and number (percent) for categorical variables. The prevalence and incidence of dyskalemia (<3.0 or >5.5 mmol/L) were calculated as the number of participants with dyskalemia divided by the relevant analytic sample. A two-sided P value of <0.05 was considered statistically significant. All statistical analyses were performed using Stata 15.0 (StataCorp, College Station, TX).

## Results

### Prevalence analyses

#### Baseline characteristics of persons with hypertension who had laboratory testing

Mean age of the 1,073 participants was 54 (SD 9.5); 71% were male (Table 1). The average systolic/diastolic BP of screening and eligibility visits was 159/96 mmHg; 59.8% had SBP 140–159 and/or DBP 90–99 mmHg, and 40.2% had SBP 160–179 and/or DBP 100–120 mmHg. Based on self-report, 16% had diabetes, but other conditions, such as heart, kidney, and lung disease and stroke, were rare (all <0.5%). For kidney function and proteinuria, the mean CrCl was 70 (SD 22) ml/min, and 97% of participants had negative dipstick proteinuria.

**Table 1:** Characteristics of 1,073 adults with hypertension in Bangladesh in community BP screening in the overall population and stratified by the status of dyskalemia.


CHARACTERISTICS	OVERALL (n = 1073)	PREVALENT DYSKALEMIA STATUS AT SCREENING		

		HYPOKALEMIA < 3 MMOL/L (n = 17)	NORMOKALEMIA 3.0 TO 5.5 MMOL/L (n = 1054)	HYPERKALEMIA* > 5.5 MMOL/L (n = 2)

Age years, mean (SD)	54 (9.5)	54 (7.9)	54 (9.5)	–

Male gender, no (%)	761 (71)	10 (59)	750 (71)	–

Height cm, mean (SD)	159 (8.9)	155 (7.3)	159 (8.9)	–

Weight kg, mean (SD)	64 (12)	60 (9.5)	64 (12)	–

Body mass index kg/m^2^, mean (SD)	25 (4.0)	25 (2.7)	25 (4.0)	–

SV Systolic BP mmHg, mean (SD)	159 (13)	169 (13)	159 (13)	–

SV Diastolic BP mmHg, mean (SD)	95 (10)	101 (13)	95 (10)	–

EV Systolic BP mmHg, mean (SD)	155 (13)	163 (14)	155 (13)	–

EV Diastolic BP mmHg, mean (SD)	93 (9.9)	98 (14)	93 (9.8)	–

Average of SV and EV – systolic BP, mean (SD)	157 (12)	166 (13)	157 (12)	–

Average diastolic BP, mean (SD)	94 (9.3)	99 (13)	94 (9.2)	–

Medical history, no (%)				

No history	891 (83)	9 (53)	881 (84)	

Heart failure	0 (0.0)	0 (0.0)	0 (0.0)	–

Myocardial infarction	1 (0.1)	0 (0.0)	1 (0.1)	–

Stroke	4 (0.4)	1 (5.9)	3 (0.3)	–

Diabetes	175 (16)	7 (41)	166 (16)	–

Kidney disease	2 (0.2)	0 (0.0)	2 (0.2)	–

COPD, Asthma	0 (0.0)	0 (0.0)	0 (0.0)	–

Have you ever smoked?, no (%)				

Never	735 (69)	13 (76)	720 (68)	–

Former	112 (10)	1 (6)	110 (10)	–

Current	225 (21)	3 (18)	222 (21)	–

Creatinine clearance (CrCl), ml/min mean (SD)	70 (22)	58 (17)	70 (22)	–

Dipstick proteinuria result?, no (%)				

Negative	1037 (97)	15 (88)	1019 (97)	–

Trace	12 (1)	0 (0)	12 (1)	–

1+	20 (2)	2 (12)	18 (2)	–

2+	3 (0)	0 (0)	3 (0)	–

3+	2 (0)	0 (0)	2 (0)	–


*Data are not shown for those with hyperkalemia due to small sample size (n = 2).

#### Prevalence of dyskalemia, hypokalemia, and hyperkalemia

**Figure 2:**
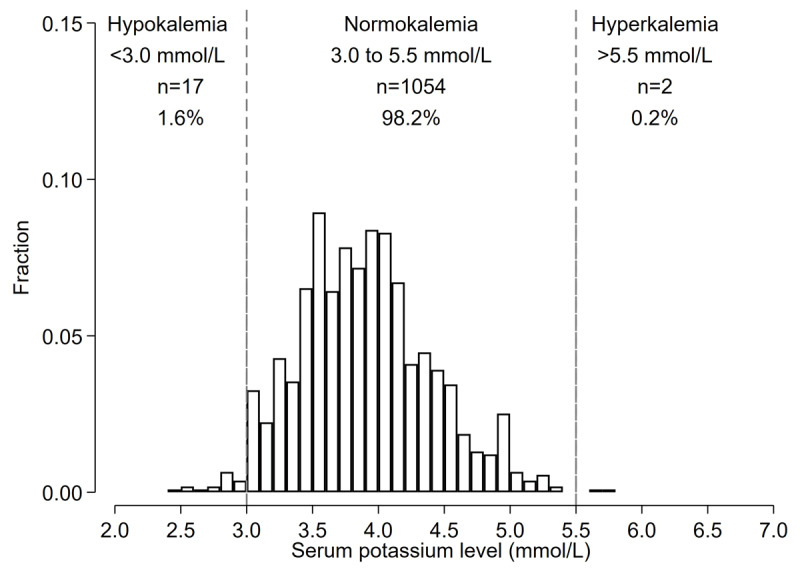
Histograms of baseline potassium levels among Bangladeshi adults with hypertension identified during screening (Prevalence analysis). *Histogram shows the distribution of potassium levels prior to the start of FDC pills among those who were candidates for starting FDC pills (n = 1,073).

Mean serum potassium was 3.9 (0.5) mmol/L, and 19 (1.8%) participants had dyskalemia as defined by K < 3.0 mmol/L (n = 17 [1.6%]) or >5.5 mmol/L (n = 2, 0.2%) (Figure 2). There were 213 participants (19.9%) with potassium levels 3.0–3.5 mmol/L and 15 participants (1.4%) with potassium levels 5.0–5.5 mmol/L.

### Incidence Analyses

#### Incidence of dyskalemia, hypokalemia and hyperkalemia after initiating FDC

**Figure 3:**
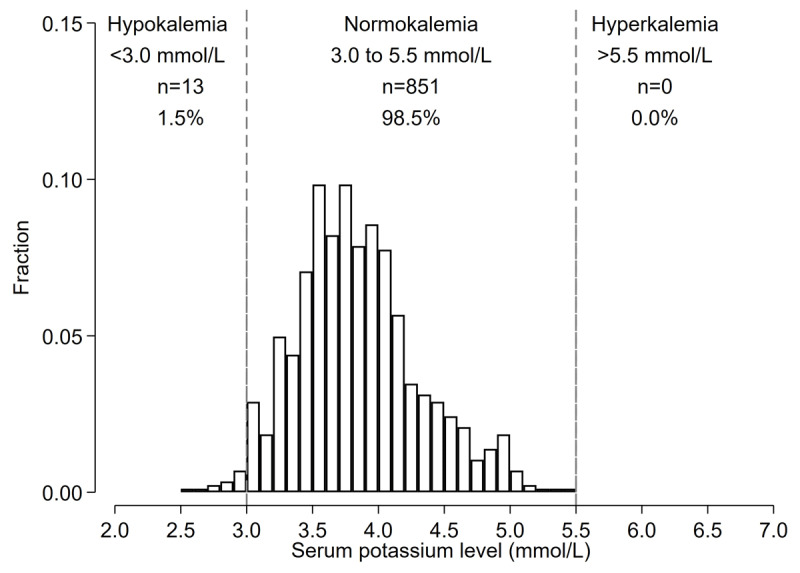
Histograms of potassium levels at follow-up among Bangladeshi adults with hypertension who started FDC (Incidence Analysis). *Among 1,017 participants who initiated FDC pills, 864 completed the follow-up and are included in the histogram.

Of the 1,073 included in the prevalence analysis, 1,017 participants initiated FDC. Reasons for not initiating FDC were K <3.0 mmol/L (n = 17), K >5.0 mmol/L (n = 17), CrCl <30 ml/min (n = 16), and not returning to the hypertension clinic to receive FDC pills (n = 6). Of 1,017 patients who initiated the study FDC, 864 participants completed the follow-up with a blood test for potassium; median number of days after FDC initiation was 36 (IQI 33–45) days. The mean (SD) change in serum potassium between before and after initiation of FDC was –0.05 (0.53) mmol/L (Figure S1). Incident dyskalemia occurred in 13 (1.5%) participants, all the cases were hypokalemia (<3.0 mmol/L) (n = 13) (1.5%), and none developed severe hypokalemia (<2.5 mmol/L). There was no incidence of hyperkalemia (>5.5 mmol/L) (Figure 3). Those who had incident hypokalemia had a mean change in potassium levels of –0.73 (0.48) mmol/L between baseline and follow-up visits (Figure S2).

#### BP change after initiating FDC

**Figure 4:**
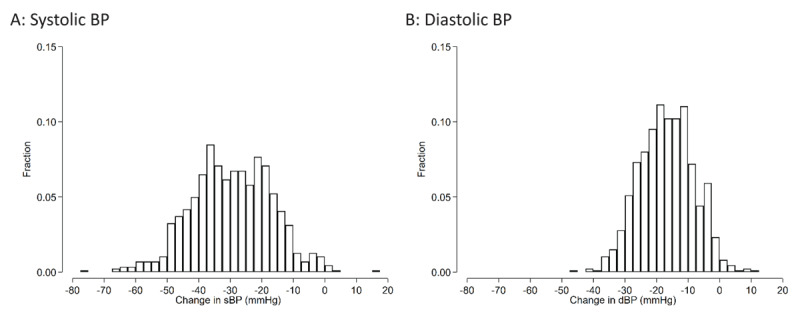
Histograms of changes in blood pressure between baseline and follow-up visits among Bangladeshi adults with hypertension who started FDC. The mean systolic BP change (95%CI) was –29.8 (–30.6 to –28.9) mmHg. The mean diastolic BP change was –16.9 (–17.5 to –16.4) mmHg.

The mean (SD) change in SBP/DBP before and after FDC initiation was –29.8 (12.7) / –16.9 (8.6) mmHg (Figure 4). There was no difference in changes in BP between those who did and did not develop severe hypokalemia (P > 0.05). 794 (91.8%) participants had an SBP <140 and DBP <90 mmHg at follow-up.

#### Self-reported adherence and symptoms at follow-up

Self-reported adherence to the medication was excellent, with nearly 94% having reported that they did not miss a dose of medication more than one day over the past seven days and last took the medication on the day of, or the day before, the blood test (Table S2). 1.3% self-reported some symptoms. All symptoms were temporary, including one who reported dizziness or lightheadedness or fainting, five who reported swelling of ankles, and three who reported weakness (Table S3). There was one sudden death of undetermined cause.

## Discussion

In this study of community-dwelling middle-aged and older adults with untreated hypertension in Dhaka, Bangladesh, the prevalence of hypokalemia (K <3.0 mmol/L) and hyperkalemia (>5.5 mmol/L) was low at 1.6% and 0.2%, respectively. After excluding those with K < 3.0 or >5.0 mmol/L or creatinine clearance <30 ml/min, at 2 months after initiating FDC of telmisartan 40 mg and amlodipine 5 mg, the incidence of hypokalemia was 1.5%, and no one developed incident hyperkalemia.

In our study, no one developed incident hyperkalemia K >5.5 mmo/L within 1–3 months after using the FDC pill, including ARB. In the ONTARGET, the incidence of hyperkalemia (K >5.5 mmo/L) at six weeks after initiating telmisartan was 1.6% (14), although the mean baseline potassium level was 4.4 mmol/L in the ONTARGET vs. 3.9 mmol/L in our study (15). Two electronic healthcare record-based cohorts from North America reported that the incidence of K >5.5 mmol/L following an initiation of RASI was 0.7% within a month (16) and 1.7% within one year (17). Low incidence of hyperkalemia in Bangladesh is possibly due to different diets, since lower dietary intake of potassium was reported in Asian populations compared to European and US populations (18). We excluded high-risk individuals (e.g., those with potassium levels >5.0 mmol/L or creatinine clearance <30 ml/min) from initiating FDC pills, which may also play a role. In the CREOLE Study (19) and a Phase II clinical trial of the single-pill combination of telmisartan and amlodipine (20), both studying unselected patients with hypertension, potassium levels were similar across randomization groups, with no reports of hyperkalemia as an adverse event.

Hypokalemia was a dominant phenotype of dyskalemia in our study even for incident cases after the use of FDC, including telmisartan. Underlying reasons for our observation are not fully clear. Some participants with hypokalemia might have other conditions that can lower potassium levels, such as primary aldosteronism, although we do not have information on plasma levels of aldosterone and renin. Our findings raise a concern about using thiazide diuretics for treating hypertension (especially without blood lab tests), either as a monotherapy or as an FDC pill in Bangladesh (21). Whether our results are consistent in other low- and middle-income countries, especially neighboring countries of Bangladesh with similar lifestyles and environments, should be investigated.

At follow-up, 91.8% of participants achieved SBP <140 and DBP <90 mmHg with our study FDC pill, in line with results from randomized trials showing the reduction of BP by FDC pills of telmisartan 40 mg and amlodipine 5 mg (11). Such findings are encouraging since early achievement of target BP (e.g., SBP <140 mmHg within 1–3 months) was shown to reduce the risk of cardiovascular disease and mortality (22). The mean reduction in SBP at follow-up was approximately 30 mmHg, which likely reflects both regression to the mean (~10 mmHg) and the blood pressure-lowering effects of the FDC (~20 mmHg) (23,24). Despite this substantial BP reduction, only a few people self-reported symptoms related to low BP, such as dizziness, lightheadedness, or fainting. Other symptoms, such as swelling of legs, which may be expected with treatment of hypertension with amlodipine, were also rare (<1%) and mild.

Our study demonstrates that after excluding prevalent dyskalemia and kidney dysfunction, the initiation of an FDC pill of telmisartan and amlodipine is safe and effective to treat adults with hypertension without monitoring serum potassium levels, at least soon after initiation of FDC. While our follow-up was limited to two months, this duration should be sufficient to detect dyskalemia associated with RASI, given the pharmacokinetics and short half-life (<2–3 days) of telmisartan and amlodipine (25). Whether blood tests are not needed for the long term is a challenging question, especially since patients’ conditions (e.g., kidney dysfunction) can change over time, albeit slowly in most individuals. In general, the National Committee for Quality Assurance recommends at least annual monitoring of serum potassium for people who are using RASI (26). However, laboratory monitoring is commonly overlooked even in high-income countries (e.g., US (27), Netherlands (28)). For example, a study from the US reported nearly one-third of patients who were on RASI did not have laboratory tests at least yearly (27).

Whether we should perform blood tests before the initiation of antihypertensive medications is another difficult issue. It is ideal to check blood tests before starting medications influencing metabolic blood markers like RASI. However, such a blood test is often not feasible in resource-limited settings. The benefit of reducing the risk of clinical events by lowering BP likely outweighs the potential harm of inducing hyperkalemia when an FDC combining amlodipine and a RASI is initiated without prior blood testing (29,30,31). However, this decision may vary depending on local contexts, such as the prevalence of hypo- versus hyperkalemia, the burden of hypertension, societal acceptance of risk, and access to blood tests.

Our study has limitations. First, although our single-arm study design was appropriate for our specific question, this design did not allow for disentangling whether the incidence of dyskalemia or self-reported symptoms were due to the FDC use or other factors unrelated to the medication. Second, several secondary outcomes, such as symptoms and medication adherence, were based on self-report, which might be subject to measurement bias. Third, we minimized in-person data collection in order to reduce the burden of study participation. Therefore, some clinical data that are commonly collected in persons with hypertension (e.g., fasting blood sugar, lipid profiles, electrocardiograms) were not obtained in this study. Nonetheless, our study has pragmatic implications, especially in resource-limited settings in low- and middle-income countries. Fourth, the retention rate was <90% despite the short study period. However, the main reasons for missing the last visit were being out of town or having a busy schedule but not clinical signs or symptoms. Finally, we followed up with our patients over 1–3 months, and thus whether and to what extent our observation stays consistent over time should be assessed in future studies.

Our study also has several strengths, including a large, diverse, hard-to-reach population and a high follow-up rate. Enrollment of a community-based sample of middle-aged and older adults with untreated hypertension from Dhaka, Bangladesh, strengthens generalizability and is particularly applicable to Bangladesh, which has initiated a major initiative to treat and control its large population with untreated hypertension. Our findings might also be generalizable to other LMICs, especially in neighboring countries with similar lifestyles and environments, where healthcare resources are limited.

In conclusion, in Bangladeshi adults with newly diagnosed hypertension, the prevalence of hyperkalemia was less than 0.5%, and we did not observe any incident cases of hyperkalemia after the use of FDC with telmisartan and amlodipine over ~2 months. Given the low prevalence and incidence of hyperkalemia and the evident reduction in BP, our study suggests initiating FDC with telmisartan and amlodipine may be a practical and safe option for newly diagnosed hypertension, especially in resource-constrained settings where blood tests cannot be easily obtained. Future research should assess the long-term impact of FDC use on serum potassium levels and consider exploring higher risk populations (e.g., individuals with potassium levels 5-5.2 mmol/L or CrCl 20–30 ml/min).

## Additional File

The additional file for this article can be found as follows:

10.5334/gh.1415.s1 Supplemental Tables and Figures. Tables S1–S3, Figures S1 and S2.
