# Supplementary material for: Dyskalemia Prior to and After Initiation of a Fixed Dose Combination of Telmisartan and Amlodipine in Adults with Hypertension in Bangladesh
Source: Glob Heart. 2025 Mar 19;20(1):30. doi: 10.5334/gh.1415 (PMC11927674; doi:10.5334/gh.1415)

Supplemental tables and figures

Table S1: Inclusion and exclusion criteria for the study

**Inclusion criterion**	**Definition**
SBP ≥ 140 mmHg or DBP ≥ 90 mmHg	SBP ≥140 mmHg or DBP ≥90 mmHg, based on the average of BP measurements taken at the screening and eligibility visits. At the screening visit in the community, we first measured BP in a sitting position without resting time, and those who had SBP ≥140 or DBP ≥90 mmHg at the second BP measurement were invited to visit the hypertension clinic. At the hypertension clinic, we first measured BP in a sitting position without resting time, and the second BP was measured after 5 minutes of rest. The average of the two BP measurements at the screening and eligibility visits was used for the inclusion criterion of SBP ≥140 mmHg or DBP ≥90 mmHg.
Age ≥ 40 years for men and ≥ 50 years for women who have been menopausal for ≥ 1 year	Men were eligible if aged ≥ 40 years. Women were eligible if aged ≥ 50 years and had been menopausal for at least 12 months, as determined by the question, "When was your last menstrual period? [< 12 months/≥ 12 months]."
Not currently taking BP medications	People reporting taking any medications for treatment of hypertension within the past month were excluded. This was determined by the question, "Have you taken hypertension medications in the past month?"
**Exclusion criterion**	**Definition**
SBP of ≥180 mmHg or DBP of ≥120 mmHg	Individuals with SBP ≥ 180 mmHg or DBP ≥ 120 mmHg at any visit (screening or eligibility visit) were excluded and referred to a hypertension clinic for care.
History of intolerance or allergy to RASI or CCB	Participants were excluded if they responded yes to the question, "Have you had a bad reaction when you took hypertension medications?"
History of serious medical conditions determined by medical officers	Participants were excluded if medical officers, during eligibility interviews, identified serious medical conditions in their medical history. These individuals were referred for care outside the study.
Creatinine clearance <30 ml/min*	Participants were excluded if creatinine clearance based on the Cockcroft-Gault equation was < 30 ml/min

Abbreviations: SBP, systolic blood pressure; DBP, diastolic blood pressure; RASI, renin-angiotensin system inhibitors. *Excluded from the initiation of the study FDC with telmisartan and amlodipine.

Table S2: Self-reported adherence to the FDC medication

Questions/Responses	Overall (n=864)		By incident dyskalemia status at follow-up			
			Severe hypokalemia < 3 mmol/L (n=13)		Mild hypokalemia, normokalemia or mild hyperkalemia 3 to 5.5 mmol/L (n=851)	
	Count	%	Count	%	Count	%
How many days did you miss a dose of meds over the past 7 days?						
0 days	798	92.4%	13	10.0%	785	92.2%
1 day	17	2.0%	0	0.0%	17	2.0%
2 days	16	1.9%	0	0.0%	16	1.9%
3 days	17	2.0%	0	0.0%	17	2.0%
4 days or more	16	1.9%	0	0.0%	16	1.9%
Total	864	10.0%	13	10.0%	851	10.0%
When was the last day you took the medication?						
Today	6	0.7%	0	0.0%	6	0.7%
Yesterday	796	92.1%	13	10.0%	783	92.0%
Day before yesterday	41	4.8%	0	0.0%	41	4.8%
3 days before	12	1.4%	0	0.0%	12	1.4%
4 days or more before	9	1.0%	0	0.0%	9	1.1%
Total	864	10.0%	13	10.0%	851	10.0%

* Data are not shown for hyperkalemia since there was no participant who developed hyperkalemia (> 5.5 mmol/L).

Table S3: Self-reported symptoms at follow-up

Questions/Responses	Overall (n=864)		By incident dyskalemia status at follow-up			
			Severe hypokalemia < 3 mmol/L (n=13)		Mild hypokalemia, normokalemia or mild hyperkalemia 3 to 5.5 mmol/L (n=851)	
	Count	%	Count	%	Count	%
Questions asked in all participants (n=864) (check boxes)						
Dizziness or lightheadedness or fainting	1	0.12%	0	0.0%	1	0.12%
Swelling of ankles	5	0.58%	0	0.0%	5	0.59%
Others (provided in free form text)		0.00%				
Burning sensation in hand and feet	1	0.12%	0	0.0%	1	0.12%
Cough	1	0.12%	0	0.0%	1	0.12%
Weakness	3	0.35%	3	23.08%	0	0.00%
Any	11	1.28%	3	23.08%	8	0.94%

* Data are not shown for severe hyperkalemia since no participant developed > 5.5 mmol/L.

Figure S1: Histograms of change in potassium levels among persons who started FDC. The mean change (95%CI) in potassium levels was -0.1 (-0.1 to -0.0) mmol/L.

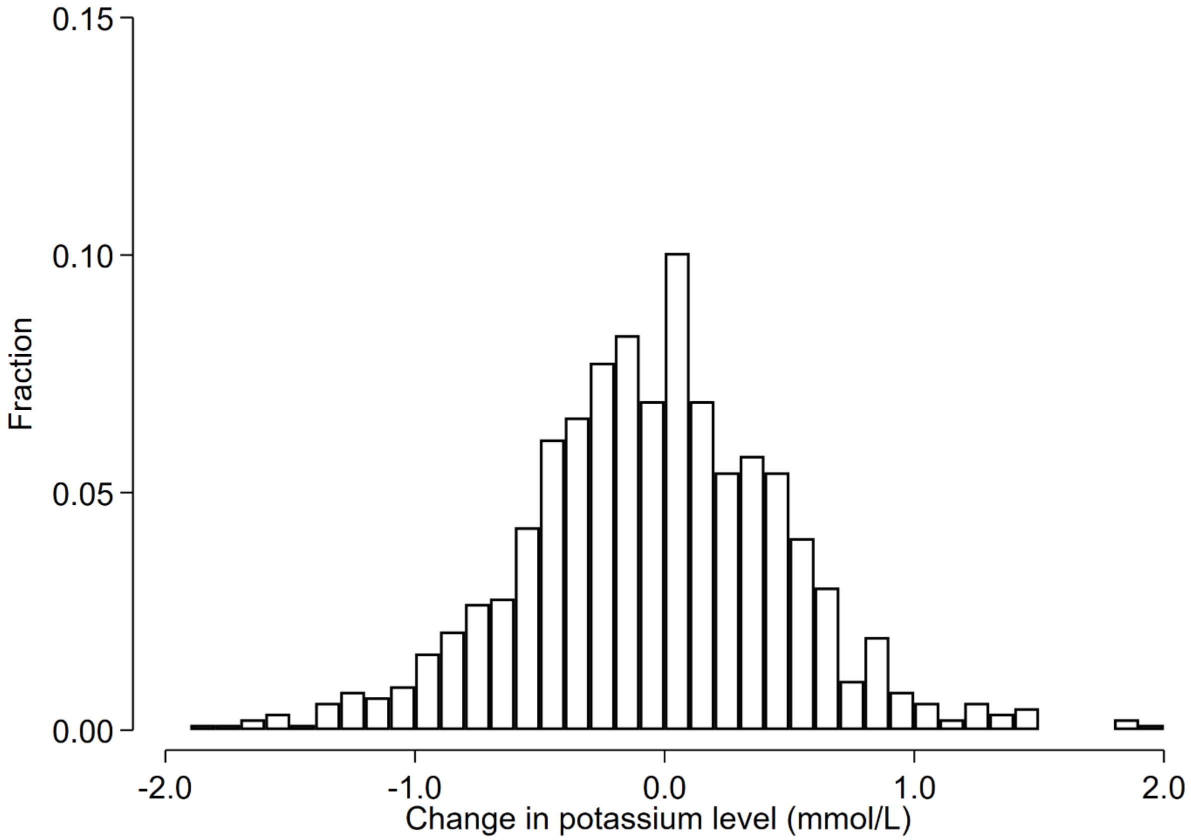

Figure S2: Spaghetti plots for change in potassium levels between baseline and follow-up visits among those who had incident hypokalemia

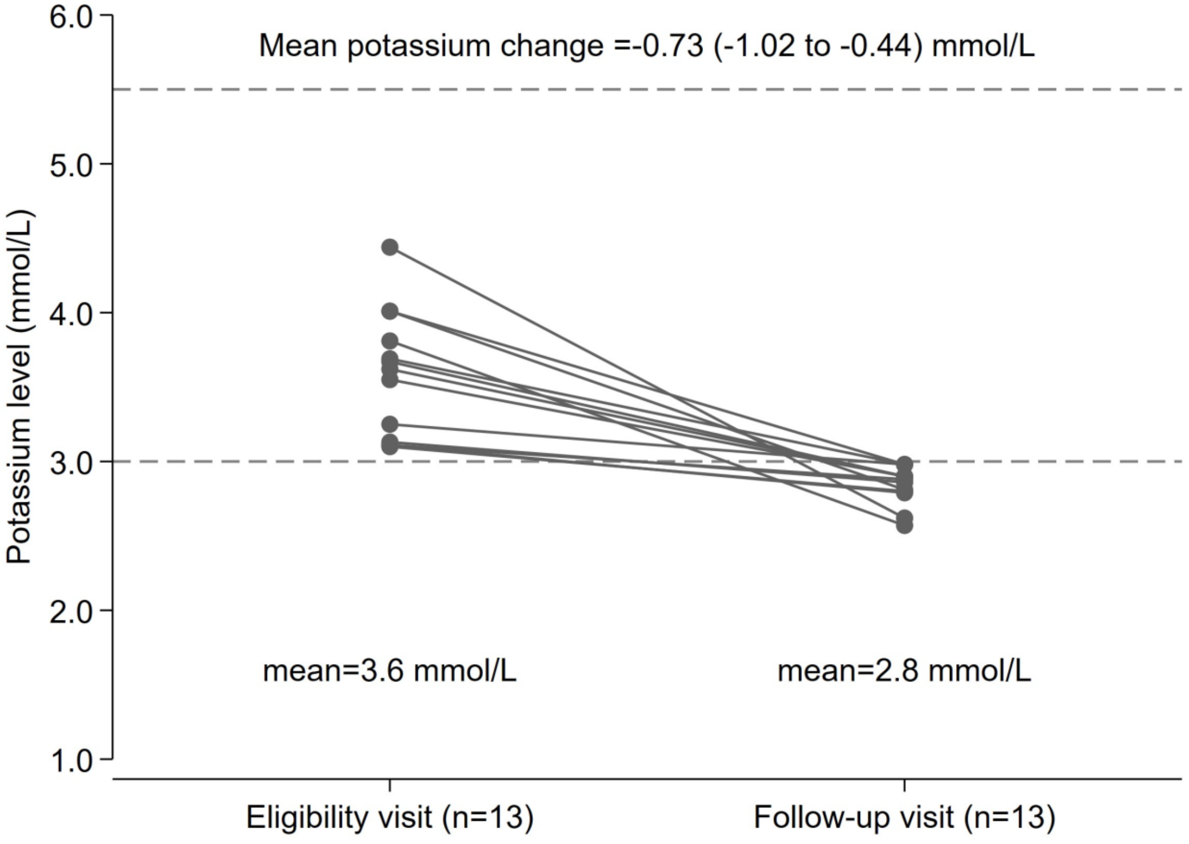

Supplement: Supplemental Tables and Figures. — Tables S1–S3, Figures S1 and S2. [file gh-20-1-1415-s1.pdf]
